# Feasibility of calculating rocuronium dosage by skeletal muscle weight in patients with obesity

**DOI:** 10.3389/fmed.2024.1399475

**Published:** 2024-05-30

**Authors:** Zhenhua Hu, Benmu Li, Zhanwen Li, Zhe Liu, Shengqun Liu

**Affiliations:** ^1^Department of Anesthesia and Perioperative Medicine, Henan Provincial People's Hospital, Zhengzhou, Henan, China; ^2^Department of Sports Medicine and Nutrition, School of Health and Rehabilitation Sciences, University of Pittsburgh, Pittsburgh, PA, United States

**Keywords:** general anesthesia, obesity, body composition analysis, rocuronium, muscle relaxation monitoring

## Abstract

This study aimed to investigate the dose–response relationship of rocuronium administered based on skeletal muscle weight and to assess the feasibility of calculating rocuronium dosage by skeletal muscle weight in short surgeries for patients with obesity. This single-center, randomized controlled clinical trial included 71 patients with obesity aged 28–70 years, with body fat percentages (PBF) >20% in men and > 28% in women, ASA status I-III, scheduled for tracheoscopy. Patients were randomly allocated into two groups: skeletal muscle group (SM group) received rocuronium based on the skeletal muscle content (1.0 mg/kg, *n* = 31), and the conventional administration group (conventional group) received rocuronium based on total body weight (0.45 mg/kg, *n* = 30). General anesthesia was administered using the same protocol. Parameters recorded included patients’ general condition, muscle relaxant usage, onset time of muscle relaxants, non-response time, clinical effect time, 75% recovery time, and recovery index. Additionally, occurrences of body movement, choking, and incomplete muscle relaxation during surgery were recorded. Compared to the conventional group, the SM group required significantly less rocuronium dosage, resulting in significantly lower non-response time, clinical effect time, 75% recovery time, and recovery index (*p* < 0.05), and the onset time is slightly longer. Neither group experienced body movement, choking, or incomplete muscle relaxation (*p* > 0.05). Utilizing skeletal muscle weight to calculate rocuronium dosage in short surgeries for patients with obesity can reduce dosage, shorten recovery time, and prevent residual muscle relaxation while achieving satisfactory muscle relaxation to meet surgical requirements.

## Introduction

Patients with obesity often exhibit an increased proportion of body fat and reduced blood flow through adipose tissue (1). When dosing anesthetic drugs based on actual body weight, the apparent volume of drug distribution and metabolic rate can be significantly impacted, affecting the quality and duration of awakening resulting in accumulation and prolonged duration in clinical anesthesia for patients with obesity (2–4). Suggestions have been made to administer drugs to patients with obesity based on their defatted body weight to adjust anesthetic dosage (5). However, previous studies diagnosing obesity using body mass index (BMI) may overlook occult obesity, where body weight appears regular but excessive fat is present in body composition analysis, thus not adequately addressing drug metabolism considerations.

Direct segmental multi-frequency Bio-impedance analysis (BIA) is utilized in body composition analyzers to accurately assess the content and proportion of body components such as muscle, fat, and water, reflecting the nutritional status, obesity, swelling, and other physical health conditions of the human body (6). The body composition analyzer calculates the defatted weight of the subject based on parameters including age, gender, height, and weight. The standard percentage of body fat (PBF) is 15% for men and 23% for women, with 10–20% for men and 18–28% for women considered normal ranges. A percentage of body fat (PBF) >20% for men and > 28% for women is indicative of obesity (7, 8). Hence, in our study, obesity is based on body fat ratio, which better represents the actual body condition than solely relying on height and weight.

Simultaneously, rocuronium is a fast-acting, medium-latency non-depolarizing neuromuscular blocking agent that exerts its effect by competitively binding to N-type acetylcholine receptors at the motor end plate. Skeletal muscle content and the number of neuromuscular junctions may influence its clinical effect, leading us to speculate on the feasibility of using skeletal muscle weight to calculate rocuronium dosage (9).

In this study, we utilized a body composition analyzer to determine the skeletal muscle content of all patients with obesity, including those with occult obesity characterized by normal BMI but excessive body fat content. This served as a reference for dosing, allowing us to observe the dose–response relationship of rocuronium administered based on skeletal muscle weight and investigate the feasibility of calculating skeletal muscle relaxants by skeletal muscle weight in patients with obesity undergoing short surgeries.

## Methods

This study, involving human subjects, was approved by The Ethics Committee of Henan Provincial People’s Hospital [2021(160)], and all patients provided signed informed consent forms.

Seventy-one patients proposed for tracheoscopy since May 2023 in Henan Provincial People’s Hospital were divided into two groups by random number table method, the skeletal muscle content administration group (SM group) and the conventional administration group (conventional group). (N1 = N2 = 2[(tα/2 + tβ/2)S/δ]2, taking α = 0.05 and β = 0.1, and using rocuronium dosage as a reference to calculate the sample size, each group needs about 28 cases).

Inclusion criteria: age 28–70 years, percentage body fat (PBF) >20% for men and > 28% for women, ASA class I-III. Exclusion criteria: abnormal liver and kidney function or disorders of water-electrolyte and acid–base balance; previous history of tuberculosis, neuromuscular disease, history of chemotherapy for oncological diseases; drugs affecting the conduction function of neuromuscular junction within the last month; expected operation time < 1 h; metal implants in the body.

After the patient was admitted to the room, monitoring the blood pressure, heart rate, pulse oximetry, and electrocardiogram. The intravenous induction of general anesthesia consisted on midazolam 0.03 mg/kg, sufentanil 0.2 μg/kg, and propofol 2–4 mg/kg. After the patient falls asleep, we performed muscle relaxation monitoring of the thumb adductor using a TOF-Watch SX muscle relaxation monitor (Organon Teknika, Netherlands). The stimulation electrodes were attached to the forearm at the location of the ulnar nerve at the wrist, with the distal electrode being placed on the radial side of the proximal flexor line through the ulnar carpal flexor and the proximal electrode 2 to 3 cm from the distal electrode. The maximum plane of the acceleration transducer was attached to the palmar root of the thumb, and the temperature sensor to the medial palmar interphalangeal area. The forearm was fixed and wrapped with gauze for insulation.

The method employed Train of Four stimulation (TOF) with specified parameters: a stimulation intensity of 50 mA, frequency of 2 Hz, pulse width of 0.2 ms, pulse spacing of 500 ms, and a stimulation interval of 12 s. The initial twitch response to the TOF was standardized to a stable 100% as the basal value (Tc). Following 3 min of continuous stimulation, rocuronium was administered intravenously. The conventional group received a dose of 0.45 mg/kg (1.5 times the ED95), while the SM group received 1.0 mg/kg based on skeletal muscle weight. For standard weight patients, approximately 45% of their total body weight was attributed to skeletal muscle, resulting in a calculated ED95 of 0.66 mg/kg administered based on skeletal muscle weight. Upon achieving a TOF ratio of 0, a laryngeal mask was placed, and mechanical ventilation initiated with specific parameters: tidal volume (VT) set at 6–8 mL/kg, ventilation frequency at 12–18 times/min, inspiratory-to-expiratory ratio (I:E) at 1:1–1:2, inhaled oxygen concentration maintained between 50 and 100%, with an inhaled oxygen flow rate of 2 L/min, and a target end-tidal CO2 (PETCO2) range of 30–45 mmHg (1 mmHg = 0.133 kPa). Anesthesia maintenance was achieved through intravenous infusion of propofol at a rate of 4 ~ 8 mg·kg^−1^·min^−1^ and remifentanil at a rate of 0.2 ~ 0.5 μg·kg^−1^·min^−1^. Intraoperative blood pressure and heart rate were monitored to ensure fluctuations did not exceed 20% of basal levels. Additional inotropic drugs were not administered intraoperatively, and postoperative inotropic antagonism was avoided.

Primary observation indexes: (1) myorelaxant dosage; (2) neuromuscular function monitoring: onset of action, the duration from the termination of drug administration to the point at which TOF stimulation elicits no response (TOF = 0); non-response time, the interval from the disappearance of the first twitch response of TOF (T1) to its reappearance; Clinical action time, the period from the conclusion of drug injection to the restoration of T1 to 25% of its baseline value; 75% recovery time, the duration between drug injection cessation and the reestablishment of T1 to 75% of its initial value; recovery index, the timeframe from T1’s recovery to 25% of the baseline value to its recuperation to 75% of the baseline value. These parameters collectively provide valuable insights into the neuromuscular effects and recovery kinetics following neuromuscular blockade.

Secondary observation indexes: (1) patients’ basic conditions: skeletal muscle mass, age, gender, BMI, body fat ratio, ASA classification, blood potassium and sodium concentration, total plasma protein, and plasma albumin levels, and operation time; (2) occurrence of body movement, choking, and incomplete muscle relaxation during the operation.

Statistical analysis was conducted using SPSS 20.0 software (SPSS Inc., Chicago, IL, United States). Normally distributed measures were presented as mean ± standard deviation (*x ± s*). Repeated measures design data were analyzed using ANOVA with repeated measures, while comparison between groups was performed using the two independent samples t-test. The comparison of count data was conducted using the *X*^2^-test. A *p*-value of less than 0.05 was considered indicative of statistical significance.

## Results

**Figure 1:**
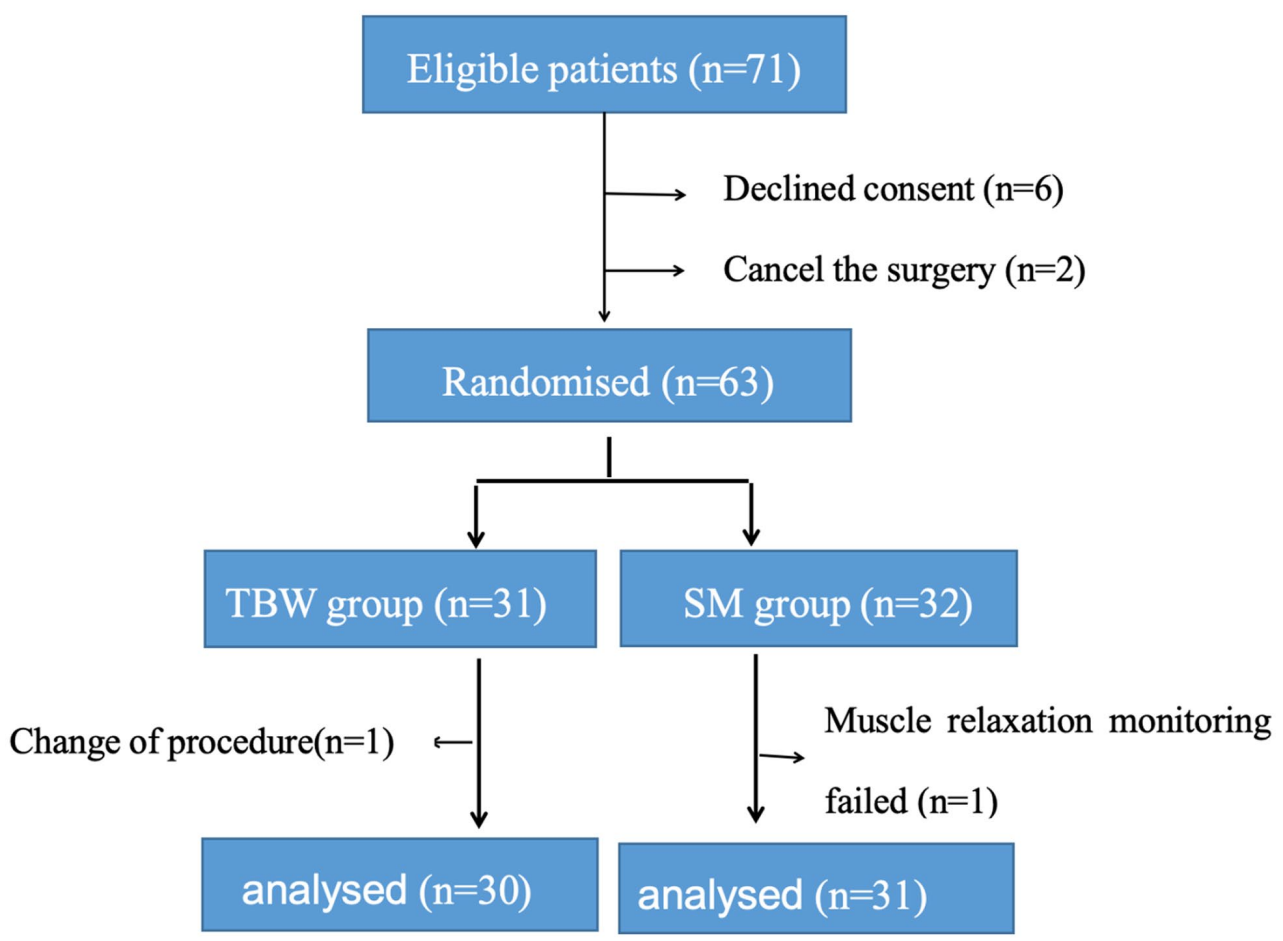
CONSORT diagram. CONSORT, consolidated standards of reporting trials.

Out of 71 screened patients, 6 declined to participate in the study, 2 had their surgery temporarily canceled, 1 underwent a change in surgical method requiring additional muscle relaxation drugs, and 1 experienced muscle relaxation monitoring failure. Ultimately, 30 cases were included in the Conventional group and 31 cases in the SM group for statistical analysis (Figure 1).

There were no statistically significant differences observed in the general condition, body composition, blood biochemistry, plasma protein levels, or surgery time between the two groups of patients (*p* > 0.05) (Table 1).

**Table 1:** Comparison of general condition and surgery time between two groups of patients (*x̅ ± s*).

Group	Conventional group (*n* = 30)	SM group (*n* = 31)	*P*-value
Gender (M/F)	20/10	18/12	0.599
Age (years)	55.73 ± 13.11	57.83 ± 11.65	0.514
Duration of surgery (min)	35.77 ± 8.73	33.68 ± 11.24	0.426
Weight (kg)	69.55 ± 11.99	72.11 ± 12.12	0.421
BMI (kg/cm^2^)	28.57 ± 3.51	27.19 ± 2.93	0.891
Skeletal muscle mass (kg)	26.67 ± 4.92	25.71 ± 6.28	0.518
Body fat percentage (%)	34.94 ± 6.02	35.35 ± 6.41	0.393
Water content (L)	35.68 ± 5.94	34.39 ± 7.61	0.475
Inorganic salt	3.30 ± 0.50	3.24 ± 0.68	0.690
Protein (kg)	9.18 ± 1.65	9.52 ± 2.06	0.492
Blood potassium (mmol/L)	3.96 ± 0.42	3.98 ± 0.31	0.875
BLOOD sodium (mmol/L)	140.14 ± 2.06	135.43 ± 2.49	0.779
Plasma albumin (g/L)	37.26 ± 4.87	35.87 ± 7.04	0.391
Total plasma protein (g/L)	62.87 ± 7.47	64.89 ± 4.87	0.232

Compared to the Conventional group, the SM group utilized significantly lower rocuronium dosages, with significantly reduced non-response time, clinical effect time, 75% recovery time, and recovery index (*p* < 0.05), and the onset time is slightly longer. Additionally, there were no instances of body movement, choking, or incomplete muscle relaxation observed in either group (*p* > 0.05) (Table 2).

**Table 2:** Comparison of the pharmacodynamic and other indices of rocuronium between the two groups (*x̅ ± s*).

Groups	Conventional group (*n* = 30)	SM group (*n* = 31)	*P*-value
Induction dose (mg)	31.37 ± 5.43	25.77 ± 6.23	<0.001
Onset time (s)	154.97 ± 24.19	174.40 ± 20.39	0.002
Non-response time (min)	21.23 ± 4.25	18.43 ± 4.48	0.018
75% recovery time (min)	31.43 ± 4.60	27.07 ± 5.28	0.001
Recovery index (min)	13.8 ± 2.18	9.43 ± 3.35	<0.001
Body movement	0	0	/
Choking	0	0	/

## Discussion

Obesity induces various pathophysiological changes in the body, affecting the pharmacokinetic properties of numerous drugs due to associated physiological alterations. Recent studies have highlighted differences in the metabolism of intravenous and inhaled general anesthetics, as well as inotropic drugs, between obese and normal-weight patients (10). Many investigations have adjusted drug dosages for patients with obesity based on ideal or lean body weight. Notably, lean body weight (LBW) has been strongly associated with increased cardiac output compared to total body weight (TBW) and ideal body weight (IBW) in patients with obesity. Furthermore, clearance of most drugs increases linearly with LBW but not with TBW (5, 11–13). TBW-based calculations may lead to prolonged duration of action for muscle relaxants, necessitating dosing adjustments based on LBW rather than TBW. While succinylcholine induction doses should be calculated conventionally to ensure adequate muscle relaxation during intubation, non-depolarizing muscle relaxants such as vecuronium bromide, rocuronium, atracurium, and cis-atracurium should be dosed according to IBW to avoid fat accumulation and insufficient muscle relaxation (14, 15).

However, existing weight acquisition methods overlook differences in body composition, including variations in body fat and skeletal muscle content among individuals of similar body weight (6). The pharmacokinetics of occult patients with obesity, characterized by high body fat and low skeletal muscle content despite a normal BMI, have not received adequate attention. Administering drugs at actual body weight may risk overdose and accumulation in this population. In our study, all patients with obesity with excess body fat content underwent body composition analysis, and rocuronium, a skeletal muscle relaxant, was dosed based on skeletal muscle weight, ensuring adequate muscle relaxation for surgery while reducing recovery time and minimizing residual muscle relaxation.

The primary characteristics such as sex ratio, age, weight, and BMI were similar between the SM and conventional groups. Likewise, body composition analysis results including body fat content, skeletal muscle mass, body water, inorganic salt, and protein content, were comparable. No differences were observed in plasma protein, blood potassium, and blood sodium levels, which could affect muscarinic drug metabolism and neuromuscular contractile function, ensuring comparability between the two groups.

Our findings revealed significantly lower rocuronium dosage in the SM group compared to the conventional group, accompanied by lower time to no response, recovery time, and recovery index in the SM group, its onset time was slightly higher, and no signs of insufficient muscle relaxation occurred in either group. Reduced muscle relaxant dosage and recovery time can mitigate postoperative neuromuscular residual effects and associated respiratory complications (16), particularly beneficial for short, minimally invasive outpatient procedures such as tracheoscopic consultation. Previous studies utilizing lean body weight for muscle relaxant dosing in patients with obesity under anesthesia have also reported reduced dosage and shorter recovery time, consistent with our findings (14, 17, 18). However, these studies primarily focused on patients with obesity with elevated BMI and did not consider those with occult obesity characterized by excess body fat content alone. Since non-depolarizing muscle relaxants like rocuronium competitively block acetylcholine’s depolarizing effect at neuromuscular junctions, skeletal muscle content and neuromuscular junction quantity may significantly influence their clinical efficacy, warranting consideration when determining dosage.

This study has certain limitations. Firstly, in the SM group, the dose administered based on skeletal muscle mass was estimated using data on body composition and pharmacokinetics in normal populations, rather than directly determined from measured actual ED95 blood concentrations. This method of estimation assumes that neuromuscular blocking agents distribute solely within skeletal muscle tissue and not within adipose tissue. However, there may indeed be some distribution within adipose tissue in reality, potentially introducing bias into the calculated ED95. Second, we only assessed clinical effect indicators of muscle relaxants in both groups and refrained from evaluating pharmacokinetic indicators to avoid additional invasive procedures.

## Conclusion

In summary, administering neuromuscular-blocking agents based on skeletal muscle weight in minimally invasive surgeries for patients with covertly obesity may reduce the required dosage, shorten patient recovery times, and mitigate residual neuromuscular blockade effects. This approach ensures satisfactory muscle relaxation while meeting surgical demands. Human body composition analysis offers a simple, non-invasive, safe, and reliable method to measure fat and skeletal muscle weight. By calculating the requirement for skeletal muscle relaxants based on skeletal muscle weight, a certain level of individualized medication can be achieved, warranting promotion to all patients requiring muscle relaxants.

## Data availability statement

The raw data supporting the conclusions of this article will be made available by the authors, without undue reservation.

## Ethics statement

The studies involving humans were approved by the Ethics Committee of Henan Provincial People’s Hospital. The studies were conducted in accordance with the local legislation and institutional requirements. The participants provided their written informed consent to participate in this study.

## Author contributions

ZH: Data curation, Project administration, Writing – original draft, Writing – review & editing. BL: Methodology, Writing – review & editing. ZLi: Conceptualization, Supervision, Writing – original draft. ZLiu: Methodology, Validation, Writing – original draft. SL: Funding acquisition, Investigation, Methodology, Project administration, Writing – review & editing.

## References

[1] Sotornik R Brassard P Martin E Yale P Carpentier AC Ardilouze J-L. Update on adipose tissue blood flow regulation. Am J Physiol Endocrinol Metab. (2012) 302:E1157–70. doi: 10.1152/ajpendo.00351.2011, PMID: 22318953

[2] Brondeel KC Lakatta AC Torres GB Hurley JJ Kunik IL Haney KF. Physiologic and pharmacologic considerations in morbid obesity and bariatric anesthesia. Saudi J Anaesth. (2022) 16:306. doi: 10.4103/sja.sja_185_22 35898535 PMC9311176

[3] Hu Z-H Liu Z Zheng G-F Li Z-W Liu S-Q. Postoperative recovery outcomes for obese patients undergoing general anesthesia: a meta-analysis of randomized controlled trials. Front Surg. (2022) 9:862632. doi: 10.3389/fsurg.2022.862632, PMID: 35965859 PMC9366090

[4] Kaye AD Lingle BD Brothers JC Rodriguez JR Morris AG Greeson EM. The patient with obesity and super-super obesity: perioperative anesthetic considerations. Saudi J Anaesth. (2022) 16:332–8. doi: 10.4103/sja.sja_235_22, PMID: 35898529 PMC9311171

[5] Ingrande J Brodsky JB Lemmens HJ. Lean body weight scalar for the anesthetic induction dose of propofol in morbidly obese subjects. Anesth Anal. (2011) 113:57–62. doi: 10.1213/ane.0b013e3181f6d9c0, PMID: 20861415

[6] Sullivan PA Still CD Jamieson ST Dixon CB Irving BA Andreacci JL. Evaluation of multi-frequency bioelectrical impedance analysis for the assessment of body composition in individuals with obesity. Obes Sci Pract. (2018) 5:141–7. doi: 10.1002/osp4.321, PMID: 31019731 PMC6469329

[7] Lee RD Nieman DC. Nutritional assessment. Boston, MA: WCB McGraw-Hill (1996).

[8] Bray GA. Contemporary diagnosis and management of obesity. Newtown, PA: Handbooks in Health Care (1998).

[9] Doo AR Lee JH Lee Y Ko S. Influence of the amount of skeletal muscle mass on rocuronium-induced neuromuscular block. Anaesth Crit Care Pain Med. (2022) 41:101086. doi: 10.1016/j.accpm.2022.101086, PMID: 35490864

[10] Dong D Peng X Liu J Qian H Li J Wu B. Morbid obesity alters both pharmacokinetics and pharmacodynamics of propofol: dosing recommendation for anesthesia induction. Drug Metab Dispos. (2016) 44:1579–83. doi: 10.1124/dmd.116.071605, PMID: 27481855

[11] Egan TD Huizinga B Gupta SK Jaarsma RL Sperry RJ Yee JB. Remifentanil pharmacokinetics in obese versus lean patients. Anesthesiology. (1998) 89:562–73. doi: 10.1097/00000542-199809000-00004, PMID: 9743391

[12] Subramani Y Riad W Chung F Wong J. Posologie optimale de propofol pour l’induction des patients obèses morbides: Une étude randomisée contrôlée comparant l’indice bispectral et une échelle de poids idéal. Can J Anesth. (2017) 64:471–9. doi: 10.1007/s12630-017-0852-x, PMID: 28243855

[13] Cortínez LI De la Fuente N Eleveld DJ Oliveros A Crovari F Sepulveda P. Performance of propofol target-controlled infusion models in the obese. Anesth Anal. (2014) 119:302–10. doi: 10.1213/ane.0000000000000317, PMID: 24977639

[14] Sakızcı-Uyar B Çelik Ş Postacı A Bayraktar Y Dikmen B Özkoçak-Turan I. Comparison of the effect of rocuronium dosing based on corrected or lean body weight on rapid sequence induction and neuromuscular blockade duration in obese female patients. Saudi Med J. (2016) 37:60–5. doi: 10.15537/smj.2016.1.14099, PMID: 26739976 PMC4724681

[15] Leykin Y Pellis T Lucca M Lomangino G Marzano B Gullo A. The effects of Cisatracurium on morbidly obese women. Anesth Anal. (2004) 99:1090–4. doi: 10.1213/01.ane.0000132781.62934.37 15385356

[16] Brull SJ Fulesdi B. Bloqueo neuromuscular residual en pacientes vulnerables: complicaciones pulmonares postoperatorias a causa de obesidad y apnea Obstructiva del Sueño. Rev Esp Anestesiol Reanim. (2019) 66:237–40. doi: 10.1016/j.redar.2019.03.005, PMID: 30922599

[17] Van Kralingen S Van De Garde EM Knibbe CA Diepstraten J Wiezer MJ Van Ramshorst B. Comparative evaluation of atracurium dosed on ideal body weight vs. total body weight in morbidly obese patients. Br J Clin Pharmacol. (2010) 71:34–40. doi: 10.1111/j.1365-2125.2010.03803.x PMC3018024 21143499

[18] Meyhoff CS Lund J Jenstrup MT Claudius C Sørensen AM Viby-Mogensen J. Should dosing of rocuronium in obese patients be based on ideal or corrected body weight? Anesth Anal. (2009) 109:787–92. doi: 10.1213/ane.0b013e3181b0826a 19690247

